# Application of flipped classroom combined with case-based learning in Introduction to Environmental Health Science

**DOI:** 10.3389/fpubh.2023.1264843

**Published:** 2023-09-15

**Authors:** You Li, Liang Cao, Huixia Zhang, Weiyi Pang, Yan Sun, Zhiyong Zhang

**Affiliations:** ^1^Department of Environmental Health and Occupational Medicine, School of Public Health, Guilin Medical University, Guilin, Guangxi, China; ^2^The Guangxi Key Laboratory of Environmental Exposomics and Entire Lifecycle Heath, Guilin, Guangxi, China; ^3^Department of Experimental Teaching Center, School of Public Health, Guilin Medical University, Guilin, Guangxi, China

**Keywords:** flipped classroom, case-based learning, Introduction to Environmental Health Science, Master of Public Health, traditional teaching method

## Abstract

**Objectives:**

To explore the effect of flipped classroom combined with case-based learning in Introduction to Environmental Health Science for the Master of Public Health (MPH).

**Methods:**

The MPH Master’s class of 2022 was selected as the experimental group at Guilin Medical University from September to December 2022, and the flipped classroom combined with the case-based learning was adopted. The class of 2021 was the control group, and we taught them with the traditional teaching method. A self-designed questionnaire and academic performance were used to evaluate the teaching effects of the two groups.

**Results:**

There was no difference in the paper score between grades 2022 and 2021, and the design question score of grade 2022 was higher than that of grade 2021. The difference was statistically significant (*p* < 0.05). The students in grade 2022 had a high overall recognition of the teaching effect of the flipped classroom combined with case-based learning in Introduction to Environmental Health Science.

**Conclusion:**

The teaching method of the flipped classroom combined with case-based learning is more suitable than the traditional teaching method in the Introduction to Environmental Health Science for MPH. It can stimulate the independent learning ability of MPH students and improve their ability to use knowledge and an innovative spirit.

## Introduction

The State Council issued “the trial rules on the professional degree of Public Health [degrees (2001) ninth],” allowing colleges and universities to recruit Master of Public Health from 2002 at the government level. Furthermore, full-time MPH enrollment began in 2009. MPH has become the focus of public health education in China in recent years. After more than 10 years of development, MPH education has achieved great-leap-forward development in China (1).

The existing education mode of MPH is rooted in academic mastery mode, showing a similar trend of “theorization,” which led to a lack of professionalism in the curriculum system and training model. The “theorization” training model could not reflect the characteristics of the MPH degree itself and would not meet the requirements of social public health for professional degrees (2). The solution is recommended as follows: the prominent feature of MPH is that it is oriented toward professional practice and pays attention to the cultivation of students in public health practice. The curriculum system should be guided by the needs of the public health profession, and the core goal is to improve students’ core professional competence. The application of flipped classrooms combined with case-based learning has achieved remarkable results in students’ practical innovation abilities for some curriculums (3, 4).

The flipped classroom (FC) is a brand-new pedagogical approach that inverts teacher-centered and lecture-based traditional education into student-centered active learning education (5). In FC progress, students’ study pre-prepared course materials pre-class without the restrictions of time and place and participate in face-to-face interactive learning and problem solving in class, often with collaborative small group activities under the instructor’s guidance (6). FC leads to a shift from passive learning to active learning, facilitates higher-order learning of the materials, and promotes the development of various cardinal skills, thus overcoming the shortcomings of traditional LBC with desired results (7). A flipped classroom model also has the potential to influence students’ attitudes toward learning and motivation, given the autonomy provided within a course (8).

Case-based learning (CBL) is a special teaching methodology of problem-based learning (PBL) that focuses on learners and guides students to learn and explore through cases. Grauer et al. (9) noted that CBL methods require less time and are more efficient in providing large amounts of material compared to PBL. In CBL, the steps are: Prior reading →Problem → Seeking out extra information → Interview with a knowledge expert (10). Meanwhile, CBL is an in-class activity that can be applied within the FC model (11, 12) where medical students work in groups to deal with health-related questions.

Case-based learning based on flipped classroom can combine the advantages of the two (13), and with network technology (such as Yu Classroom, Mu Classroom, QQ, or Wechat Platform) as the carrier, we provide targeted guidance to students before, during, and after class to achieve the purpose of curriculum reform.

Introduction to Environmental Health Science is one of the required courses for the Master of Public Health (MPH). It is a new subject that has developed gradually in the process of human adaptation to the environment. Therefore, our research adopted the method of combining flipped classroom and case-based learning in the teaching of Introduction to Environmental Health Science for MPH to improve the theoretical level of environment and health of MPH students, their independent learning ability, and their innovative spirit and achieved a good teaching effect.

## Methods

### Participants

From September to December 2022, a cluster sampling method was used to select 22 MPH students from Guilin Medical University in the experimental group and 21 MPH students from 2021 in the control group. The teachers, the syllabus, and the total number of class hours (27 class hours, 1.5 credits) were identical between the experimental group and the control group. The numbers of male and female students in the experimental group and the control group were 6/15 and 11/11, respectively.

### Study design

We used traditional multimedia courseware combined with blackboard teaching methods to explain theoretical knowledge [the teacher made teaching related content (PowerPoint, PPT), explained in the class according to the order of teaching, and taught important content or difficult to understand content combined with blackboard teaching methods] for the control group. The flipped classroom combined with the case-based learning method was adopted to carry out the teaching reform for the experimental group.

According to the requirements of the syllabus and the content of the MPH’s curriculum, we have selected the classic relevant cases. There were five parts, including environmental pollution, indoor air pollution, occupational poisoning or injury, food poisoning and transgenic food, and cases of social factors’ influence on health. We selected the classic cases for each part and sent them to the students through QQ or the Rain class platform before class. Students were divided into 3–4 people per group to analyze the case and report it in class after making a slide. After the report, teachers and students would analyze and explain the relevant contents together.

## Observation indicators

### Questionnaire survey

For the experimental group, we used the self-designed ‘Introduction to Environmental Health Science Questionnaire’ to conduct a survey. After the course, questionnaires were distributed through the questionnaire star, and questionnaires were filled out in anonymous form. A total of 22 questionnaires were sent out in this study, and 22 were effectively recovered, with an effective recovery rate of 100%.

### Evaluation of academic performance

We evaluated the grade based on daily performance (30%) and paper results (70%). The difference was that the daily performance of the control group was mainly based on attendance and mainly determined by the daily reports and discussions of the experimental group. The daily reports referred to the teacher sending relevant topics and reference materials to students based on the course content before class, with 3–4 students/group reviewing and organizing the materials, making PPTs, and reporting in class. The final grade was 100, including analysis questions (75 points) and design questions (25 points).

### Statistical analysis

SPSS28.0 software was used for statistical analysis, and the measurement data were expressed as (χ- ± s). The comparison between the two groups was conducted by two-independent sample T-test. The count data were represented by *n* (%), the test level α = 0.05, and *p* < 0.05 was considered statistically significant.

## Results

### Comparison of academic performance between the experimental group and the control group

Compared with the control group, the final exam score of the control group was higher than that of the experimental group (*p* < 0.05). There was no statistically significant difference between the two groups for the paper score, and the design question score of the experimental group was higher than that of the control group (*p* < 0.05), as shown in Table 1.

**Table 1:** Comparison of academic performance between the experimental group and control group (χ- ± s).

Group	The final exam score	The paper score	The design question score
Experimental group	82.20 ± 3.47	79.67 ± 4.50	21.33 ± 1.28
Control group	85.75 ± 3.24	79.65 ± 4.60	19.65 ± 0.88
|t|	3.388	0.012	4.941
*P*	0.002	0.991	0.000

### Evaluation of the teaching effect of students in the experimental group on introduction to environmental health science

The students in the experimental group had high overall satisfaction with the teaching effect of the flipped classroom combined case teaching method applied in Introduction to Environmental Health Science. Only one student was dissatisfied with the satisfaction of classroom interaction, while the others were satisfied, as shown in Table 2.

**Table 2:** Evaluation of students’ teaching effect on introduction to environmental health science *n* (%).

Items	Very satisfied	Satisfied	Averagely satisfied	Not at all satisfied
Are you satisfied with the ‘case + flipped classroom’ format of Introduction to Environmental Health Science?	10 (45.46)	8 (36.36)	4 (18.18)	0
Are you satisfied with the assigned teaching content and hours of Introduction to Environmental Health Science?	12 (54.54)	7 (31.82)	3 (13.64)	0
Are you satisfied with the classroom atmosphere of Introduction to Environmental Health Science?	13 (59.09)	7 (31.82)	2 (9.09)	0
Are you satisfied with your academic load in Introduction to Environmental Health Science?	9 (40.91)	7 (31.82)	6 (27.27)	0
Are you satisfied with the evaluation system of Introduction to Environmental Health Science?	10 (45.45)	9 (40.91)	3 (13.64)	0
Are you satisfied with the teacher’s learning guidance method?	12 (54.55)	9 (40.91)	1 (4.54)	0
Are you satisfied with your learning motivation?	12 (54.55)	8 (36.36)	2 (9.09)	0
Are you satisfied with your self-learning ability?	12 (54.55)	6 (27.27)	4 (18.18)	0
Are you satisfied with classroom participation and interaction?	12 (54.55)	7 (31.82)	2 (9.09)	1 (4.54)

### The learning effect of experimental group students on their self-perception of introduction to environmental health science

Fifteen students (68.18%) believed that this course was better than other courses, while seven students (31.82%) believed that it was like other courses, no one thought it was worse than other courses.

### Favorite learning guidance methods for experimental group students

Among the preferred guidance methods for students, 12 liked face-to-face tutoring (54.54%), nine selected online answering questions (40.91%), and one person chose another (4.54%).

### The focus of teaching reform in the introduction to environmental health science

Seven students (31.82%) each chose these two options. One was the teaching content shifting from basic theories to cutting-edge developments in the subject, and the other was traditional teaching methods shifting to modern teaching methods, as shown in Table 3.

**Table 3:** Key content of teaching reform for introduction to environmental health science.

Item	*n* (%)
Changes in teaching methods, shift from teacher centered to student centered	5 (22.73)
Changes in teaching methods, transition from traditional teaching methods to modern teaching methods	7 (31.82)
The transformation of teaching content from basic theories to cutting-edge developments in this discipline	7 (31.82)
The change in teaching evaluation methods, from focusing on result evaluation to combining result process evaluation	2 (9.09)
Other	1 (4.54)

### Experimental group students’ understanding of blended learning

Twenty-two students (100%) believed that the blended learning model was beneficial for the learning of this course.

## Discussion

### Teaching characteristics and the current situation of introduction to environmental health science

MPH degree education is the main channel for cultivating high-level applied public health talents in China and has made significant contributions to promoting the construction of a public health talent team (14).

Introduction to Environmental Health Science, as one of the core and backbone courses of the MPH, mainly includes four modules: environment and health, occupational and health, food nutrition and health, and social behavior and health. Among them, the environment and health module mainly focuses on the relationship between the natural environment, living environment, and population health, revealing the occurrence and development patterns of environmental factors on population health and identifying, evaluating, utilizing, or controlling various environmental factors related to population health. The occupational and health module mainly focuses on the impact of working conditions on the health of occupational groups, exploring the improvement of working conditions, creating a safe, healthy, and comfortable working environment, and improving the health level of occupational groups. The food, nutrition, and health module mainly focuses on nutrients, natural active substances, dietary patterns and health, nutritional epidemiology, nutritional metabolomics, nutritional intervention strategies for different populations guided by theoretical models, microbial and chemical contamination in food, and risk assessment. Social behavior and health mainly take the health ecology model as the framework, understand the determinants of health and health promotion strategies, master the behavior ways of change of individuals, interpersonal relationships, organizations, communities, and other different levels, and apply this theory to the design, implementation, and evaluation of health promotion projects.

Because Introduction to Environmental Health Science is a compulsory course revised to adapt to the development of public health master’s education in the new situation, there are no ready-made textbooks to refer to, only corresponding teaching requirements and objectives. This requires us to continuously explore the teaching content and methods according to the requirements. Therefore, according to the characteristics of this course, we adopt the mode of the flipped classroom combined with case analysis for teaching.

### The implementation effect of joint case-based learning in the flipped classroom

By using combined case-based learning with flipped classroom, there was no significant difference between the test group and the control group in terms of MPH test scores, and the final score was lower than the control group. The possible reason was that the assessment methods of the two grades’ daily scores were inconsistent. The experimental group’s daily performance was composed of attendance, and group reporting, and discussion (the composition of reported performance: students were grouped according to the case provided by the teacher for data review, PPT production, reporting, and display), while the control group only was evaluated their daily performance based on attendance, which was more practical and considers process evaluation. Therefore, the experimental group’s daily performance was lower than the control group’s. The score of design questions in the experimental group was higher than that in the control group, which indicated that compared with the traditional teaching method, the combined case-based learning with flipped classroom had more comprehensive knowledge and enhanced students’ scientific thinking ability, which might be related to the improvement of students’ learning initiative through the combined case analysis method in flipped classroom, which had similar results with other studies. The case-based learning based on the flipped classroom was used for teaching different majors and different courses, such as ophthalmology and competitiveness-based UG curriculum (11, 15). We have achieved good results in improving students’ learning initiative, teaching satisfaction, and overall abilities.

### Students’ satisfaction with the joint case-based learning in the flipped classroom

The total satisfaction of students with the teaching form, teaching content, and class hours, classroom atmosphere, academic burden, evaluation system, and learning guidance method, autonomous learning ability, learning enthusiasm, and participation in interaction adopted by the Introduction to Environmental Health Science exceeded 85%, which was consistent with Kolahdouzan et al.’s (16) teaching reform of surgery through the use of the case-based learning of the flipped classroom, and the improvement of students’ learning satisfaction. There were many teaching contents in Introduction to Environmental Health Science. If only flipped classroom or case-based learning was used for teaching, the class hours were not enough, so we combined case-based learning with flipped classroom. Before the start of the course, relevant case content would be posted to students through the established QQ platform. Students were required to group together before the class, analyze the case based on their knowledge, and form a PowerPoint presentation or discussion to form opinions from each group. Students would report in class, and the teacher would explain the knowledge points related to the case based on the students’ reports, and the students would discuss and summarize them. Sending students the latest cutting-edge developments related to course knowledge after class, allowing them to understand the latest developments, was beneficial for expanding their research thinking and knowledge, which was the difference between graduate teaching and undergraduate teaching. This was consistent with the summaries of other scholars (1, 17) on public health education.

### The preferred guidance methods for students and the focus of teaching reform

The most popular guidance methods for students were face-to-face tutoring and online answering of questions, which indicated that students were satisfied with the mixed online and offline teaching methods and could accept new teaching modes. Students believed that the focus of teaching reform was mainly on the transformation of teaching content from basic theories to cutting-edge developments in the subject and changes in teaching methods. The introduction of cutting-edge knowledge in the discipline was mainly presented through case studies and sent to students through the QQ platform after class. This was in line with some scholars (18) who believed that using case-based learning in MPH professional teaching could build a bridge between campus and social public health needs. They hoped to develop and apply case-based learning to train students to use theory to solve practical problems and then move from practice to theory. The concept of promoting talents in the field of public health to better serve human health is consistent.

Therefore, in order to achieve the goal of MPH training, we have combined the flipped classroom and case-based learning to carry out the teaching reform of the Introduction to Environmental Health Science and achieved good results. The overall recognition of students was also high. Our result is similar to the following results. One is that using HyFlex learning for clinical learning in undergraduate clinical education during the COVID-19 era, the students had positive feedback about the HyFlex simulation learning method in acquiring knowledge and improving objective achievement (19). The other is that the integration of scenario-based simulations in radiology education led to a positive impact on learning outcomes, formative interactive learning, and filling the gap between theory and practice (20).

In future teaching, we hope to gradually improve the relevant teaching cases, improve students’ practical ability and innovative scientific research thinking, and make due contributions to the construction of a healthy China (21).

### Future prospectives and limitations

Further teaching reforms are needed in future for MPH graduate students to continuously observe the effectiveness of the reforms. The corresponding case library can be used online for everyone to learn together, which will have a better effect.

Our study had certain shortcomings that needed to be investigated further and addressed in future research. Firstly, the control group did not conduct a questionnaire survey on students. Secondly, there are fewer MPH students studying the course each semester, and it is necessary to conduct the teaching reform of the course among the MPH students recruited each year in the future.

## Data availability statement

The raw data supporting the conclusions of this article will be made available by the authors, without undue reservation.

## Ethics statement

Written informed consent was obtained from the individual(s) for the publication of any potentially identifiable images or data included in this article.

## Author contributions

YL: Conceptualization, Funding acquisition, Visualization, Writing – original draft. LC: Data curation, Formal analysis, Investigation, Methodology, Writing – review & editing. HZ: Data curation, Methodology, Writing – review & editing. WP: Project administration, Resources, Supervision, Writing – review & editing. YS: Methodology, Project administration, Software, Writing – review & editing. ZZ: Conceptualization, Funding acquisition, Supervision, Visualization, Writing – review & editing.

## Funding

The author(s) declare financial support was received for the research, authorship, and/or publication of this article. This study was supported by Guangxi Degree and Postgraduate Education Reform Project (Grant no. JGY2021128), and Guangxi Science and Technology Major Special Project (Grant no. GKAA22096026).

## Conflict of interest

The authors declare that the research was conducted in the absence of any commercial or financial relationships that could be construed as a potential conflict of interest.

## Publisher’s note

All claims expressed in this article are solely those of the authors and do not necessarily represent those of their affiliated organizations, or those of the publisher, the editors and the reviewers. Any product that may be evaluated in this article, or claim that may be made by its manufacturer, is not guaranteed or endorsed by the publisher.
